# Sport club dropout under COVID-19 in the Netherlands: do characteristics of the neighbourhood matter?

**DOI:** 10.3389/fspor.2023.1168608

**Published:** 2023-06-21

**Authors:** Remco Hoekman, Malou Grubben, Gerbert Kraaykamp

**Affiliations:** ^1^ Department of Sociology, Radboud University, Nijmegen, Netherlands; ^2^ Mulier Institute, Utrecht, Netherlands

**Keywords:** neighbourhood, sport club participation, physical environment, sport facilities, COVID-19, social inequalities, social environment

## Abstract

Sport is considered important to mitigate social and health problems related to the COVID-19 pandemic and thus contributes to a resilient society. Because of poverty, caring responsibilities, social isolation and/or health issues, caused or reinforced by the COVID-19 pandemic, (too) high thresholds may be experienced lowering sports club participation. In this article, we study dropout from sports club membership among the Dutch population in COVID times and relate it to neighbourhood characteristics to determine whether inequality in sports behaviour is increasing or decreasing. Specifically, we analyse changes in the association to sport clubs by utilizing membership register data of the National Sport Federation in the Netherlands (NOC*NSF). This longitudinal information on 3.6 million club members in 2019 within Dutch sport federations was used to analyse developments at the individual level between 2019 (pre-COVID) and 2021. Based on register information on the area of residence of sporters, neighbourhood characteristics were added to these individual membership data. Our results display that the socioeconomic status of a member's neighbourhood and the sport infrastructure in this neighbourhood impacts the likelihood of dropping out of a sport club of both youths and adults during the COVID-19 pandemic. Dropout among members is lower in higher socioeconomic status neighbourhoods and in neighbourhoods with abundant sport facilities. Remarkably, the impact of these living environment features seems higher for youth than for adults. To conclude, our study enhances a further understanding of inequalities in sport club membership dropout during COVID-19. First, it may inform policy makers to intensify sport promotion policies and to especially support sport clubs in lower status neighbourhoods. Second, given the relatively high dropout rates during the COVID-19 pandemic particular attention for retention seems necessary.

## 1. Introduction

The social and political significance of sport has grown considerably in the past decades. Within the European Union this is emphasized in the revised European Sports Charter (1) in which is stressed that sport contributes to the UN's Sustainable Development Goals. It is for expected positive social, health, educational and cultural consequences that central and local governments invest in sport and engage in facilitating a country's sport landscape. Sport clubs are, in the words of Yves Le Lostecque (Head of the Sport Unit of the European Commission), “at the heart of the sport system” (2: p. v) and in all European countries sport clubs are supported by governments. This support is mostly financial to stimulate the use of sport facilities resulting in accessible and affordable sport club participation (3). The Netherlands is one of the countries where voluntary sport clubs (being volunteer administered sport clubs) hold a central position in governmental policies due to the high share of sport club members among its population and the societal value and policy relevance ascribed to voluntary sport clubs. Active sport club participation is sometimes even embraced as a policy tool to advance healthy and resilient individuals (4, 5).

Due to the recent COVID-19 pandemic the beneficial aspects of sport (club) participation for the individual and society even gained further attention. A particular concern in this regard relates to the existing inequalities in sport (club) participation prior to COVID-19 (see for example 6–10), and the fear that COVID-19 has further enlarged inequalities (11, 12). In COVID times not participating in sports is sometimes associated with a higher risk of experiencing profound consequences after an infection (13). Moreover, reports of loneliness, anxiety, stress and insomnia increased during the COVID-19 pandemic (14–16). Sport club participation has the potential, offering a physical activity in an organized setting, to reduce some assumed negative consequences of this pandemic (17–20). Hence, governments and scientific scholars highlight the importance of upholding existing sport club participation patterns. Several studies have, however, shown that sport (club) participation rates have declined as a result of the COVID-19 restrictions (21–23). In addition, studies that focused on differences in sport participation by educational attainment or financial deprivation concluded that existing inequalities even increased (23–25). Other studies however did not find evidence for grown inequality in sport participation (26). Overall, previous studies are inconclusive on how sport club membership has developed over the course of the COVID-19 pandemic, and whether inequality therein has grown.

The current article builds on this previous rather ambivalent work and examines individual's sport club membership in the Netherlands. The Dutch case is an interesting one, as sport club membership in the Netherlands is among the highest in Europe (21). In addition, sport club membership is one of the key indicators related to sport policy in the Netherlands^1^, and valued for its social and health benefits also during the COVID-19 pandemic. Furthermore, combatting inequalities in sport club membership, amongst others by educational level and household financial position, is one of the main policy objectives of the National Sports Agreement issued in 2018 (27). Additionally, this National Sports Agreement provides a template for various local sports agreements at the municipal level. The intention of local sports agreements is to establish cooperation with local stakeholders, such as voluntary sport clubs, policy makers, and businesses, to enable a focus on neighbourhoods in which sport (club) participation levels are relatively low.

This local and neighbourhood-oriented approach fits well within a socioecological contextual theoretical rationale (28, 29). More specifically, this presumes that individual behaviour is impacted by various environments, such as family, peer group, school, and neighbourhood. Earlier research in the Netherlands already found evidence for neighbourhood effects (social and physical environment) on sport participation (7). This study showed that a favourable social environment is related to higher weekly and monthly sport participation and that a high variety of sport facilities increases the chances for monthly sport participation.

In our case, the focus is on contextual influences regarding sport club participation. We focus on the social environment and physical environment of the neighbourhoods where sport clubs (members) are located. Differences between neighbourhoods result in variation in opportunity structures, resources, and sporting habitus for the individual. For example, in neighbourhoods with a less favourable social environment, represented by the socioeconomic status of a neighbourhood, it is assumed that the sporting habitus is somehow limited, and people are less socialized with sport club participation. Consequently, sport club participation is less embedded in individual's internalized system of dispositions, or in other words in their habitus (30). Therefore, people in lower status or deprived neighbourhoods are expected to feel less urged to continue sport club participation during COVID-19. Furthermore, in deprived neighbourhoods the physical environment is less activity-friendly and has a limited variety of sport facilities, limiting the residents' opportunities to continue participating in sport during COVID-19 (31–33). In addition, previous studies have highlighted that vulnerable groups in society, amongst other people from lower socioeconomic strata, are more likely to be impacted by crises, intensifying inequalities (34, 35). For instance, because a lack of abundant resources, amongst others financial or motivational resources, may be a barrier to continue sport club participation during COVID-19. Based on a socio-ecological model for sport participation (7), we study contextual influences focusing on the within-individual development of sport club membership in different neighbourhood contexts. Accordingly, we anticipate that features of both the social and physical environment explain differences in an individual's life course in sport club participation, more specifically dropout during COVID-19. We more specifically investigate to what extent the socioeconomic composition of sport club member's neighbourhood is important in explaining dropout in club membership, and how meaningful sport facilities and alternatives in the public space within the neighbourhood are.

Compared to previous studies on social inequality in sport participation during COVID-19 we aim to make several advancements. First, we specifically focus on sport club participation during COVID-19, with its societal relevance, instead of the wider concept of individual sport participation. We do this for both the young (4 till 18 years) and adult (older than 18 years) population in the Netherlands. Second, while most previous studies utilize cross-sectional data, a strong suit of our research is that we use longitudinal register data of 2019 and 2021 on within-individual development in sport club membership in the Netherlands. The year-to-year register data of sport club memberships is comparable at the individual level and identifies intra-personal changes in sport club membership between 2019 and 2021. This enables us to study individual dropout over these years with great detail. With this we can add to existing qualitative studies (e.g. 36), or cross-sectional approaches (e.g. 23). Third, we build on earlier research by including environmental (neighbourhood) aspects into an explanatory model for dropout during COVID-19. Most previous studies understandably focused on the impact of individual characteristics on sport participation during COVID-19. As the Knowledge Information System Sport (KISS)-data of NOC*NSF on club membership offers accurate information on where sport club members live, we can enrich individual data with neighbourhood characteristics and information on the physical environment. With this, we intend to provide a more complete picture of the role of the physical environment (e.g., sport facilities, sport infrastructure) and social environment (socioeconomic status of the neighbourhood) in explaining differences in sport club dropout during the COVID-19 pandemic. To sum up, our research question reads: *To what extent does a neighbourhood's socioeconomic status and sport infrastructure impact the likelihood for youths and adults to dropout of sport club membership in the Netherlands during the COVID-19 pandemic?*

## 2. Theoretical lens

Our socio-ecological approach is grounded in the idea that not one factor or set of factors adequately explains social behaviour (37). To understand behaviour, explanations at various contextual levels are required (38, 39). Several studies support this socio-ecological approach as described by Bronfenbrenner for the study of sport participation as affected by the social and physical environment (7, 39). Hence, our theoretical lens is a socio-ecological perspective which takes environmental systems into account and the related influence on individual behaviour. The main idea underlying Bronfenbrenner's socio-ecological model is that individuals are closely related to and influenced by their environment. Bronfenbrenner pre-dominantly argues that individual behaviours may be understood by looking at four surrounding systems: the micro-, meso-, exo- and macro-systems. These different systems may be seen as nested layers (like a set of Russian dolls), with the innermost layer representing ego. Coleman (40) underlined as well how individuals acting rationally are influenced by their environment.

First, the micro-level is made up of a complex of close relations, for example, those with family members, at the workplace, in class at school, in the neighbourhood and with one's peers. The meso-system represents the second layer. It is the context in which the micro-systems interrelate, such as the family home, the neighbourhood, and the school. The meso-system, thus, refers to relationships between micro-systems. The meso-system can be defined as the social environment, including among other things, neighbourhood socioeconomic status as one of the aspects that is assumed to influence behaviour (41, 42).

The exo-system is the third layer and refers to support settings in which individuals are not active participants. Exo-systems affecting sport participation include formal settings and physical attributes, such as sport facilities, parks, recreation centres, sport clubs and community centres. These physical attributes have been well covered in research on physical activity, particularly linking these physical attributes to physical activity and obesity (for an overview see 43). Sport-related studies have similarly sought to link the supply of sport facilities in a neighbourhood to sport participation data. As such, the presence and variety of sport facilities can be considered relevant exo-system variables in the socio-ecological model for sport club participation.

The fourth and outermost layer of Bronfenbrenner's model is the macro-system, defined as consistencies in the form and content of the lower order systems (micro-, meso- and exo-) at the level of society as a whole. Accordingly, the macro-system may not be perceived as a specific environmental context. Rather, it entails the overarching ideology, values and customs of cultures and societies, as well as general national socioeconomic and cultural conditions.

Applying the socio-ecological framework to sport behaviour requires that an individual's sport participation be seen not solely as the product of personal factors, but be linked to the environment, both social and physical, in which an individual lives. Sport behaviour is viewed as determined by multiple influences at the personal, social, physical, policy and economic levels. Most prior research on sport has focused on individual characteristics, neglecting the properties of the social, physical and policy environments in which an individual operates. For our paper we focused explicitly on the social and physical environment to identify the contextual influence on sport club dropout.

We anticipate that individuals living in a neighbourhood with a favourable socioeconomic status had more opportunities to remain a member of a sport club. Thus, we expect the dropout to be highest among individuals living in the lower socioeconomic status neighbourhoods. Regarding the physical environment of a neighbourhood, we expect that a higher score regarding sport facilities provides more opportunities to remain a member of a sport club, as this indicates the availability of more types of sport clubs and possibilities for instance to switch from indoor sports to outdoor sports. Contrary, we expect that a higher score on the neighbourhood indicator for physical activity friendly environment provides more opportunities for alternative ways of being active after people became aware of the threats of the COVID-19 pandemic. Consequently, we expect a negative relation between dropout and amount of sport facilities in a neighbourhood, and a positive relation between dropout and a more physical activity friendly environment. We formulated the following expectations: Sport club membership dropout is, (1) higher in neighbourhoods with a lower socioeconomic status; is (2) lower in neighbourhoods with ample sport facilities; and is (3) higher in neighbourhoods with a physical activity friendly environment.

With our study we contribute to the small yet growing body of knowledge on the impact of COVID-19 for social inequality in sport participation. More specifically, we attempt to fill a substantial gap in the literature on dropout in sport club membership under COVID-19. In doing so, the COVID-19 pandemic may be observed as a natural experiment that illustrates how people respond differently to a disruptive crisis, with several restrictive measures, in upholding or ending their sport club participation.

## 3. COVID-19 measures in the Netherlands

In the Netherlands as well as in other countries the COVID-19 pandemic led to serious restrictions to practice sport, also related to the sport club setting. The COVID-19 measures in the Netherlands started in March 2020 with the closure of sport facilities and stopping of sport competitions. From end of April 2020 onwards sport club activities outside were only allowed for children under the age of twelve, although no competitions or matches with other sport clubs were possible. All indoor sport club activities were still cancelled at this time. From May 2020 adults (18 years and older) were allowed to practice sport outside keeping 1,5-meter distance. From June 2020 13–18 year olds were allowed to practice sport outside without 1,5-meter distance restrictions. From July clubhouses of sport clubs were allowed to open again and practicing sports indoor was allowed as well; indoor and outdoor sports became possible for all age groups without distancing. End of September clubhouses needed to close again, and no spectators were allowed at the sport clubs venues. Sport activities itself could still take place. In October, a new lockdown was initiated, and all amateur sport competitions were stopped again. Adults were allowed to practice sport individual in public space, while youth was allowed to have team training within a sport club. All indoor sport facilities were closed. Additional measures were taken in November with only individual sport activities with a maximum of two people keeping distance. Only in March 2021 the opportunities to practice sport in sport clubs increased. For outdoor sport practices people up to 26 years old were allowed to practice sports with their own team at the outdoor sport facility. From April 2021 onwards also people over 26 years old were allowed to practice sport with a maximum of four people keeping distance. From May onwards indoor sport facilities could open conditionally. As of June, it was for adults possible to practice sport within a group and the youth was allowed to play sport matches to other clubs. Clubhouses could also open. Due to another rise of the pandemic from November onwards adults needed a COVID-19 check app to proof that they were vaccinated or had recovered from COVID-19 to get access to sport facilities. Due to a partial lockdown amateur sport activities were not allowed between 17h00 and 5h00. Indoor sport facilities were closed. In December adults were only allowed to practice sports outside alone or with one other person keeping distance. Youth could practice sports outside in larger groups and play matches, but only within their own sport club. Clubhouses needed to be kept closed. From January 2022 onwards indoor and outdoor sports was possible again, including competition matches, and clubhouses opened.

The above illustrates the barriers that members of sport clubs faced during the COVID-19 pandemic with several periods in which practicing sport at the sport club was not allowed. These restrictions could, amongst others depending on features of the social and physical environment, lead to dropout.

## 4. Material and methods

### 4.1. Data

In line with the socio-ecological theoretical model, we focus on the extent to which dropout in sport clubs during COVID-19 is explained by individual and neighbourhood features. To answer our research question, we used high-quality register information on sport club memberships in the Netherlands: Knowledge Information System Sport (KISS)-data of NOC*NSF. Information on individual sport club membership is annually provided by all Dutch sport federations that are affiliated with NOC*NSF and included in the KISS-dataset. The KISS-data provide a unique identifier for each member and insight in individual's age and the number of sport club memberships within Dutch sport federations in 2019 (pre-COVID) and 2021 (during COVID). In addition, the six-digit postal code of individual members is available which makes the connection to neighbourhood features possible. Unfortunately, no information on gender, educational level, household income or other background variables is available. We focus our analysis on membership dropout and follow individuals being a member in 2019 to their membership status in 2021 (dropout or not). The KISS data contains the full population of over 4.2 million members representing all people aged 4 years and older being a member of a Dutch sport club in 2019 (prior to COVID-19).

Based on six-digit postal codes, neighbourhood characteristics are added to the KISS-dataset. Data on the socioeconomic status of neighbourhoods were available from Statistic Netherlands (44) at postal code level. We also added information on the physical environment regarding sport opportunities in the area people live. For this we used a key indicator on general sport facilities in neighbourhoods, and an indicator of the physical activity friendliness of a neighbourhood, both made available by the Mulier Institute for the year of 2021. For more information on the calculation of these neighbourhood measures we refer to Van der Poel and colleagues (45) and Prins and colleagues (46).

### 4.2. Measurements

Within the KISS-data information was provided on a member's unique identifier, age (4–13 years, 13–18 years, 18–25 years, 25–35 years, 35–45 years, 45–55 years, 55–65 years, 65–75 years, 75 years and older), six digit postal code of home address, the number of club memberships in 2019, and number of memberships in 2021. In this article we focus on dropout and consequently selected individuals with a membership in 2019 (*N* = 4.242.668). We deleted missing cases listwise in case of a missing on place of residence or on contextual information (14.8% missing^2^), resulting in a dataset of over 3.6 million members (*N* = 3.614.875). Individuals who were registered as a sport club member in 2019 but hold no sport club membership in 2021, are considered to have completely dropped out during COVID-19.

Our contextual data is available at the neighbourhood level and is merged with the KISS-data with the use of neighbourhood identifiers. The socioeconomic status (SES) of a neighbourhood is obtained from Statistic Netherlands (SES-WOA 44); and holds information on financial welfare, educational level, and recent employment history for (almost) all households in the Netherlands. The most recent publicly available information is from 2019. The overall SES-WOA score, and the sub scores of financial welfare, educational level and employment history are calculated by Statistics Netherlands with the use of MCA (44). For reasons of interpretation, we constructed quintile scores with the bottom quintile consisting of the lowest SES neighbourhoods (1), and the top quintile representing the highest SES neighbourhoods (5).

Regarding the physical environmental context, we employed neighbourhood scores on key indicators related to sport infrastructure and sport opportunities provided by the Mulier Institute. The key indicator on physical activity friendliness of a neighbourhood (KIPAF) refers to the opportunities to be physically active in the area of living, whereas the key indicator of sport facilities in a neighbourhood (KISF) indicates the opportunity structure in terms of available sport clubs. A high score on the physical activity friendly environment means a larger variety of opportunities to be physically active in public spaces outside the sport club context. A high score on the general sport facilities indicator refers to a larger availability of sport clubs, and smaller travel distances to activities of sport clubs. Again, for reasons of interpretation quintiles were calculated for both key indicators.

To provide a strong test with respect to the role of neighbourhood features we also control for level of urbanity. In a prior scoping review, the urban environment was found to be a constraint for physical activity during COVID-19 (47). In our study level of urbanity was determined by address-density of a municipality. The address-density classification is based on the average number of addresses within 1 kilometre radius. In line with the classification of Statistic Netherlands we used five categories of urbanity: not urbanized (fewer than 500 addresses per square km (0), hardly urbanized (500–1.000 addresses per square km) (1), moderately urbanized (1.000–1.500 addresses per square km) (2), strongly urbanized (1.500–2.500 addresses per square km) (3), and extremely urbanized (with more than 2.500 addresses per square km) (4).

### 4.3. Analytic strategy

**Figure 1:**
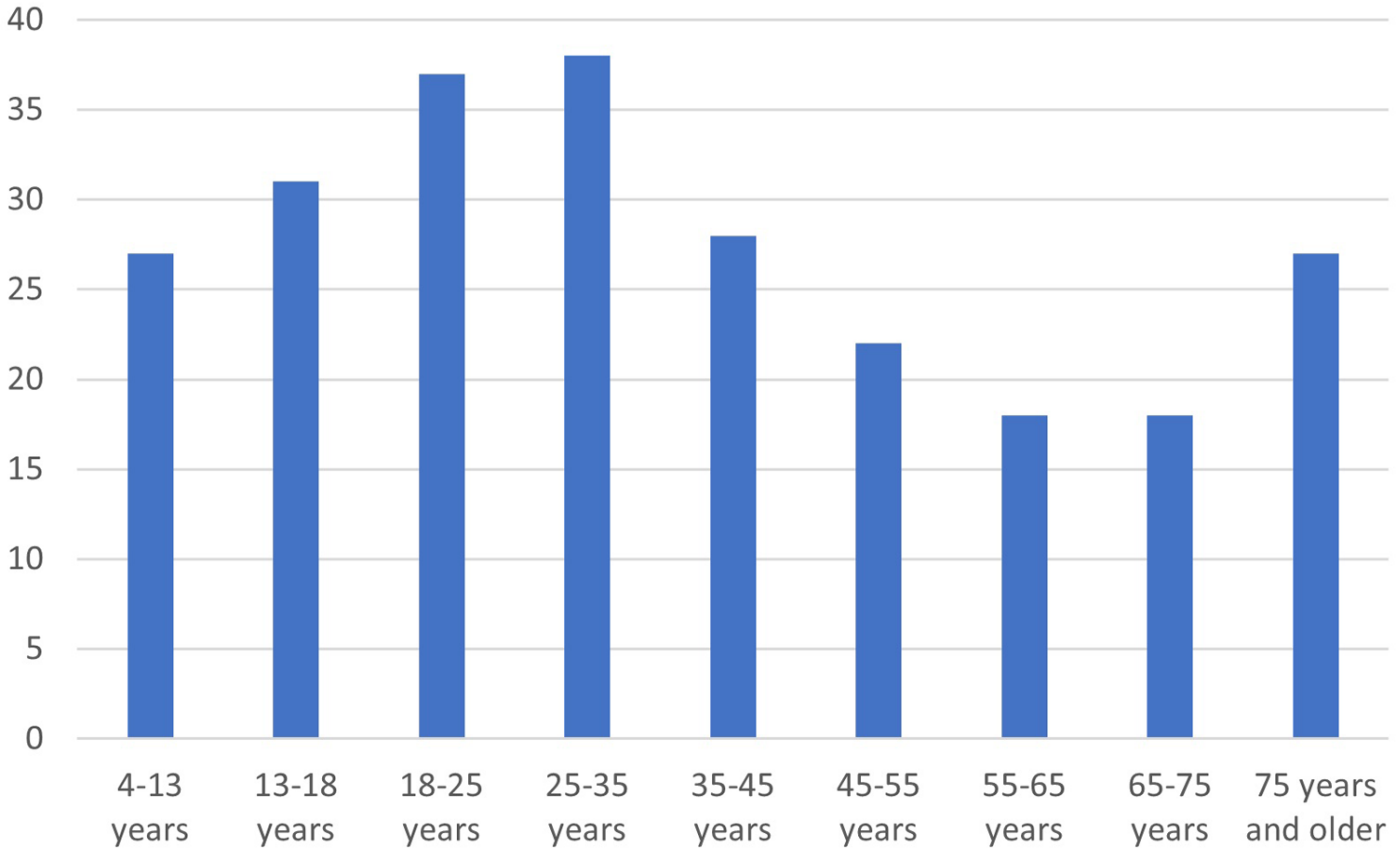
Dropout between baseline (2019) and 2021, by age (in percentages).

**Figure 2:**
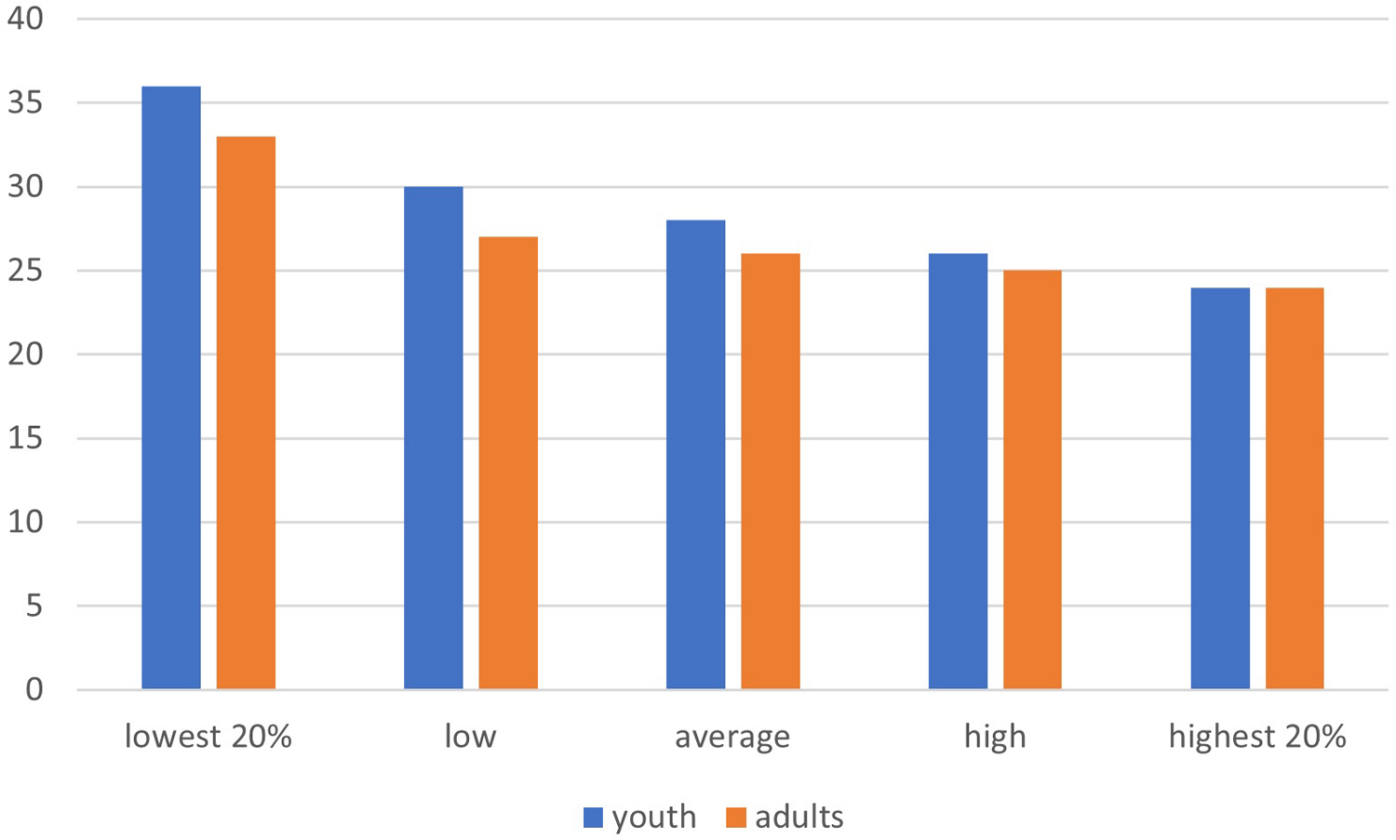
Dropout between baseline (2019) and 2021, by SES neighbourhood for youth and adults (in percentages).

**Figure 3:**
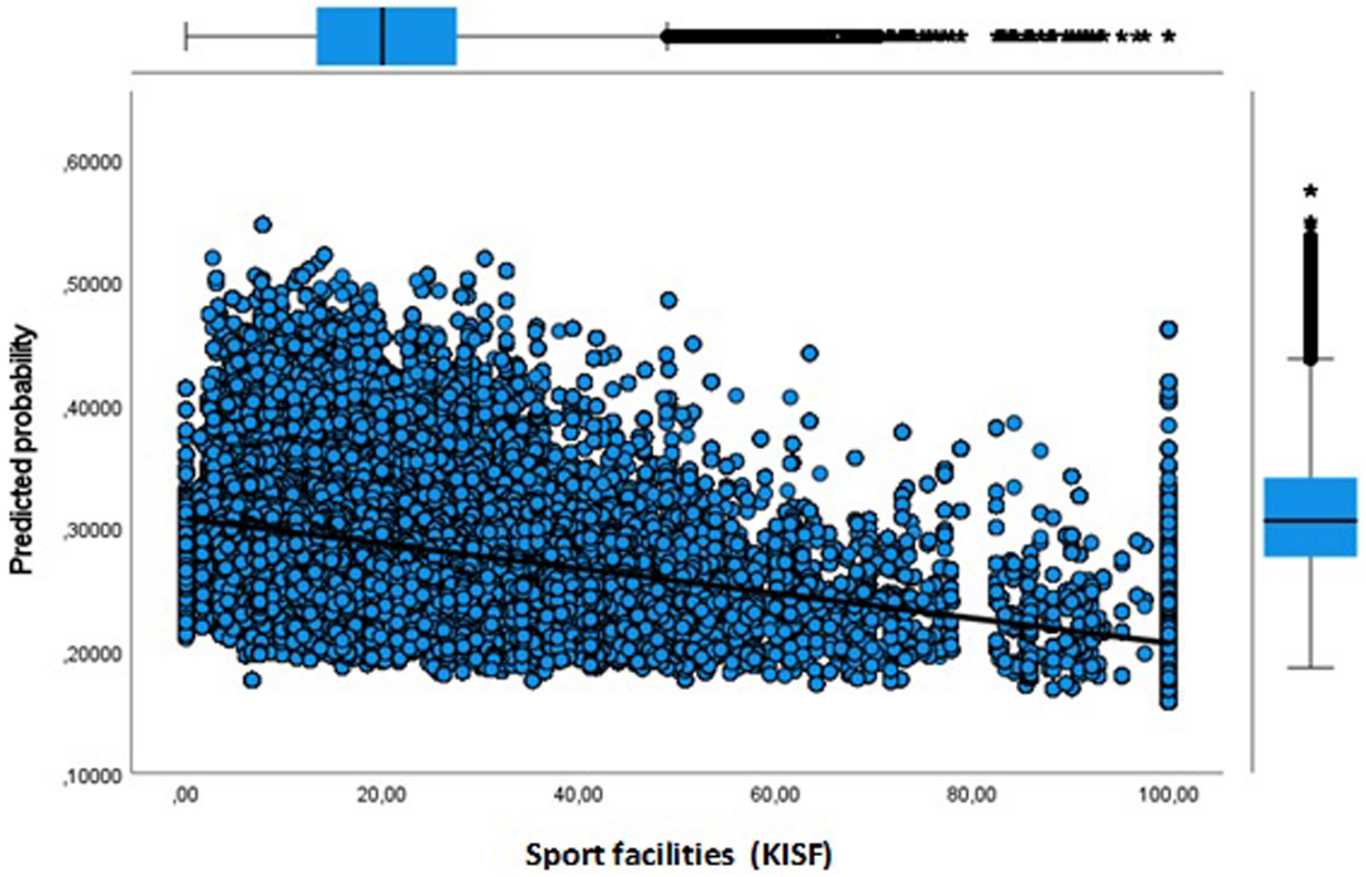
Youth predicted probability dropout by key indicator sport facilities.

**Figure 4:**
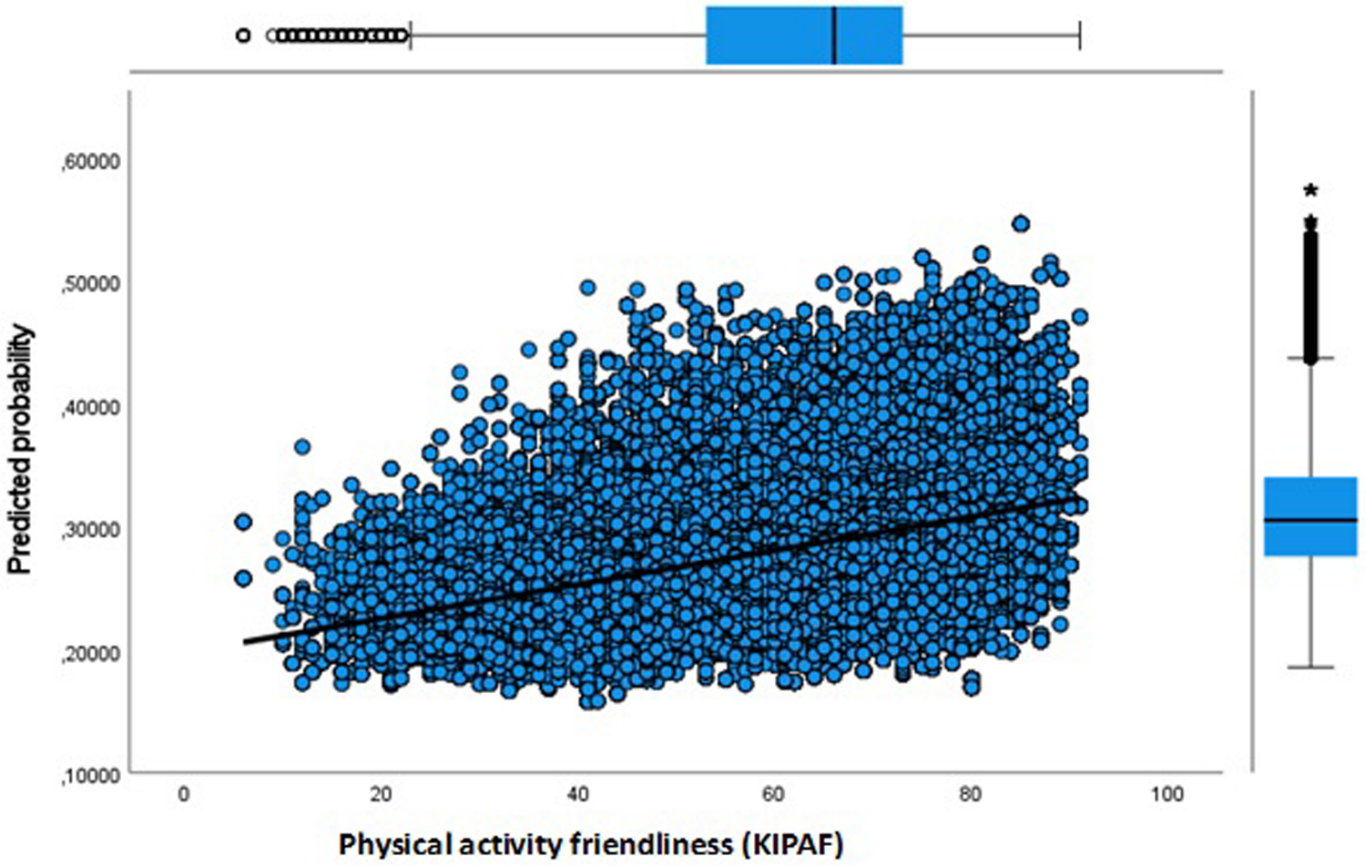
Youth dropout predicted probability by key indicator physical activity friendly environment.

**Figure 5:**
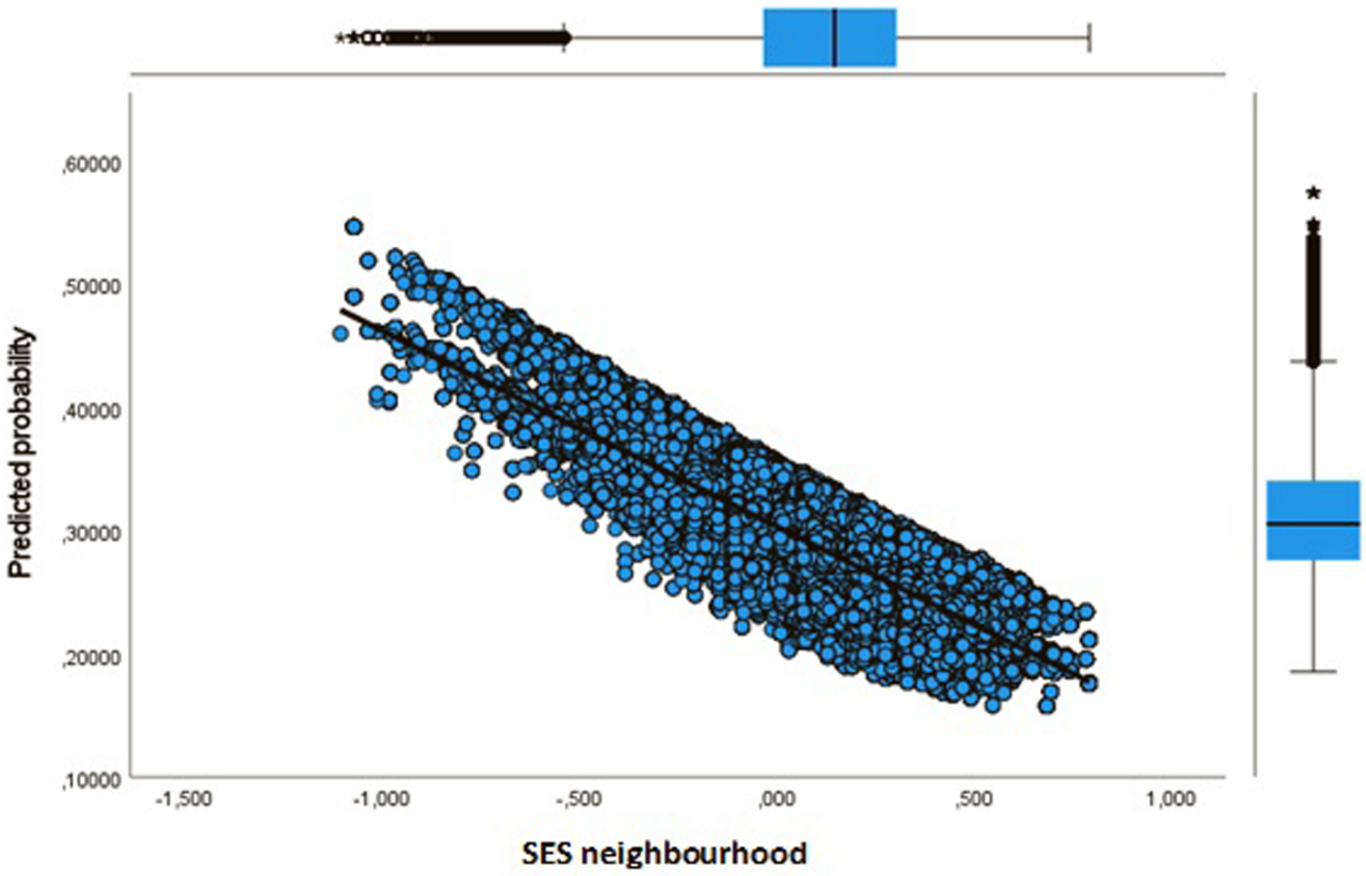
Youth dropout predicted probability by socioeconomic status of neighbourhood.

**Figure 6:**
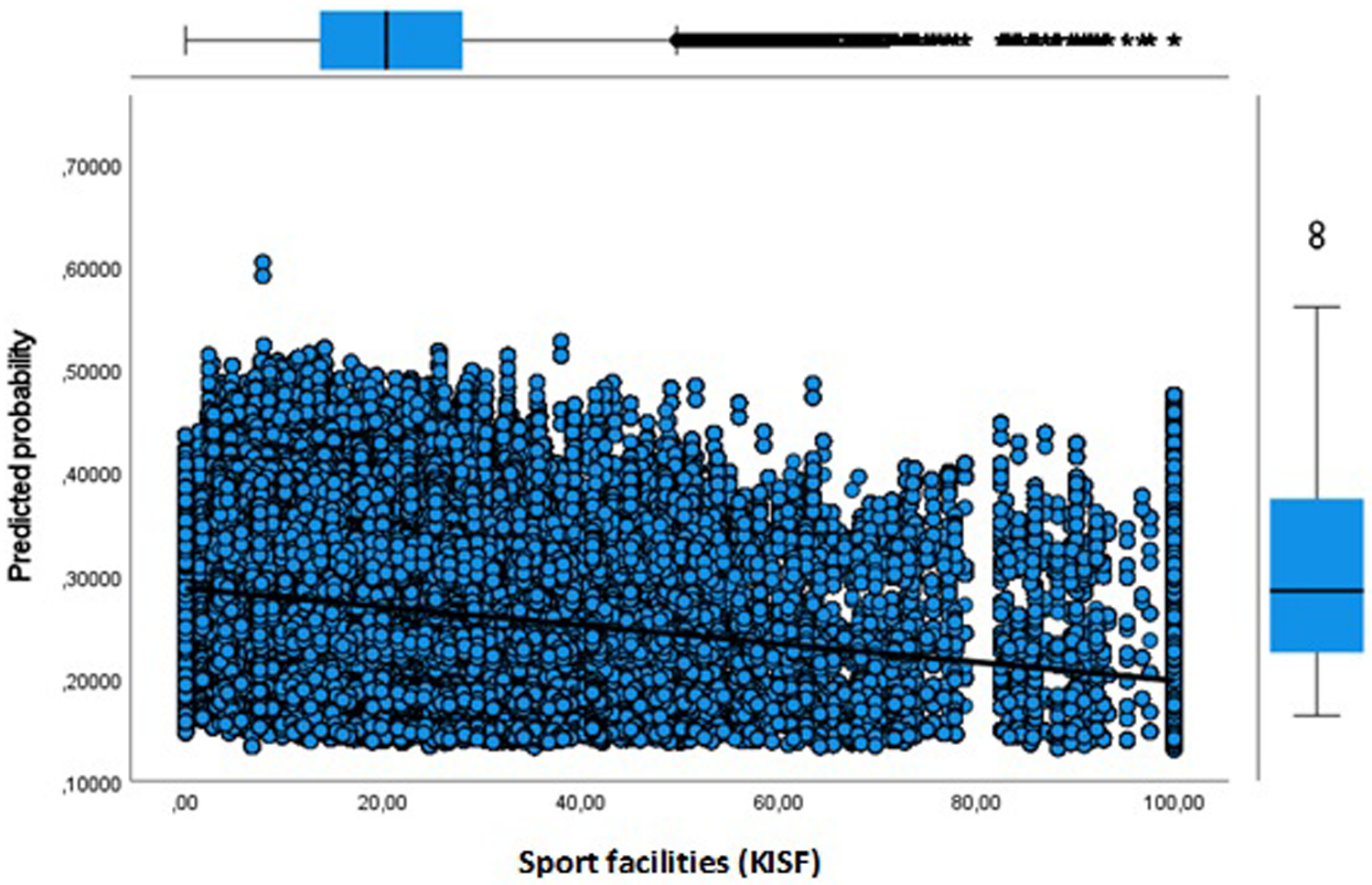
Adult dropout predicted probability by key indicator sport facilities.

**Figure 7:**
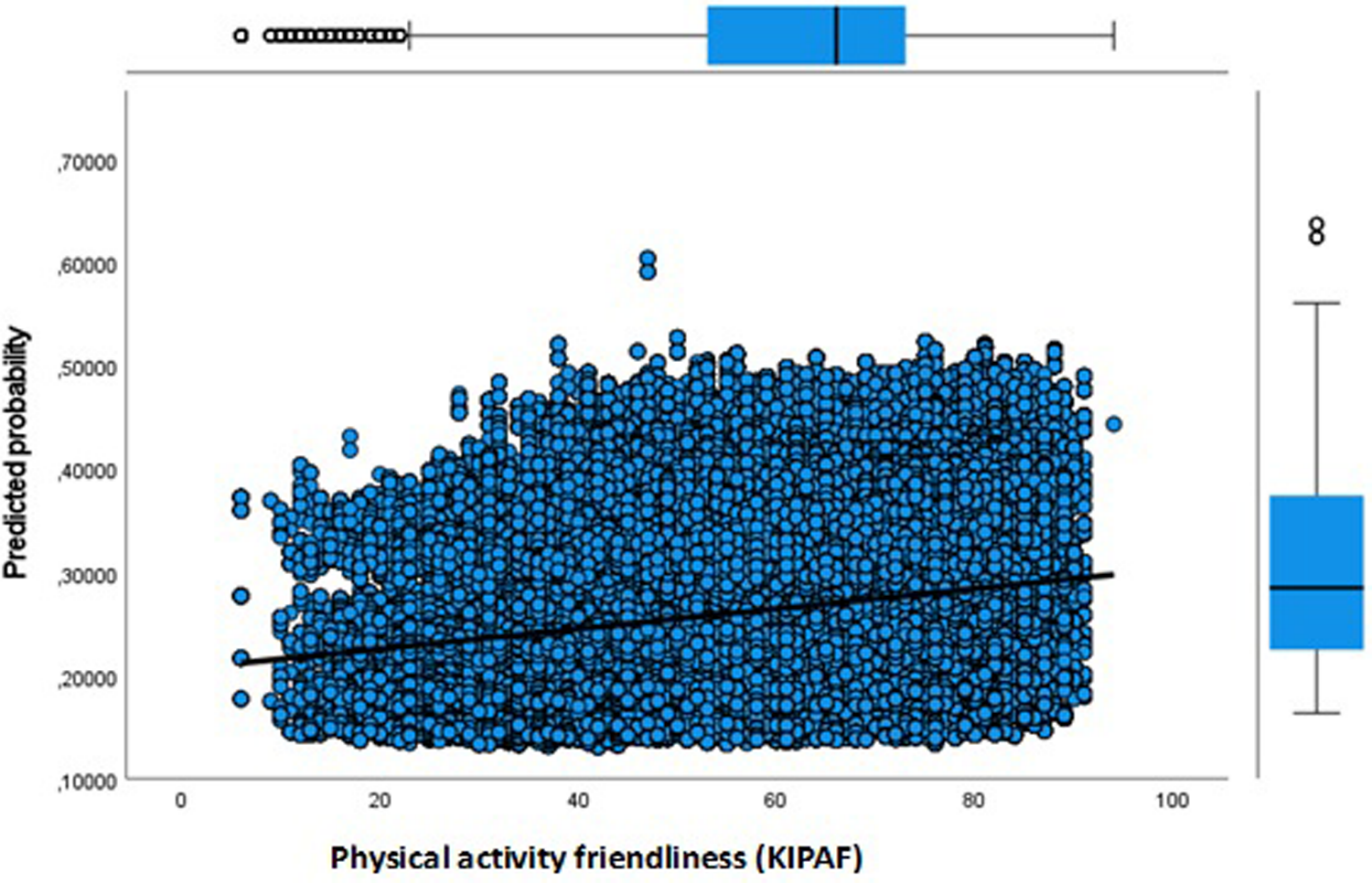
Adult dropout predicted probability by key indicator physical activity friendly environment.

**Figure 8:**
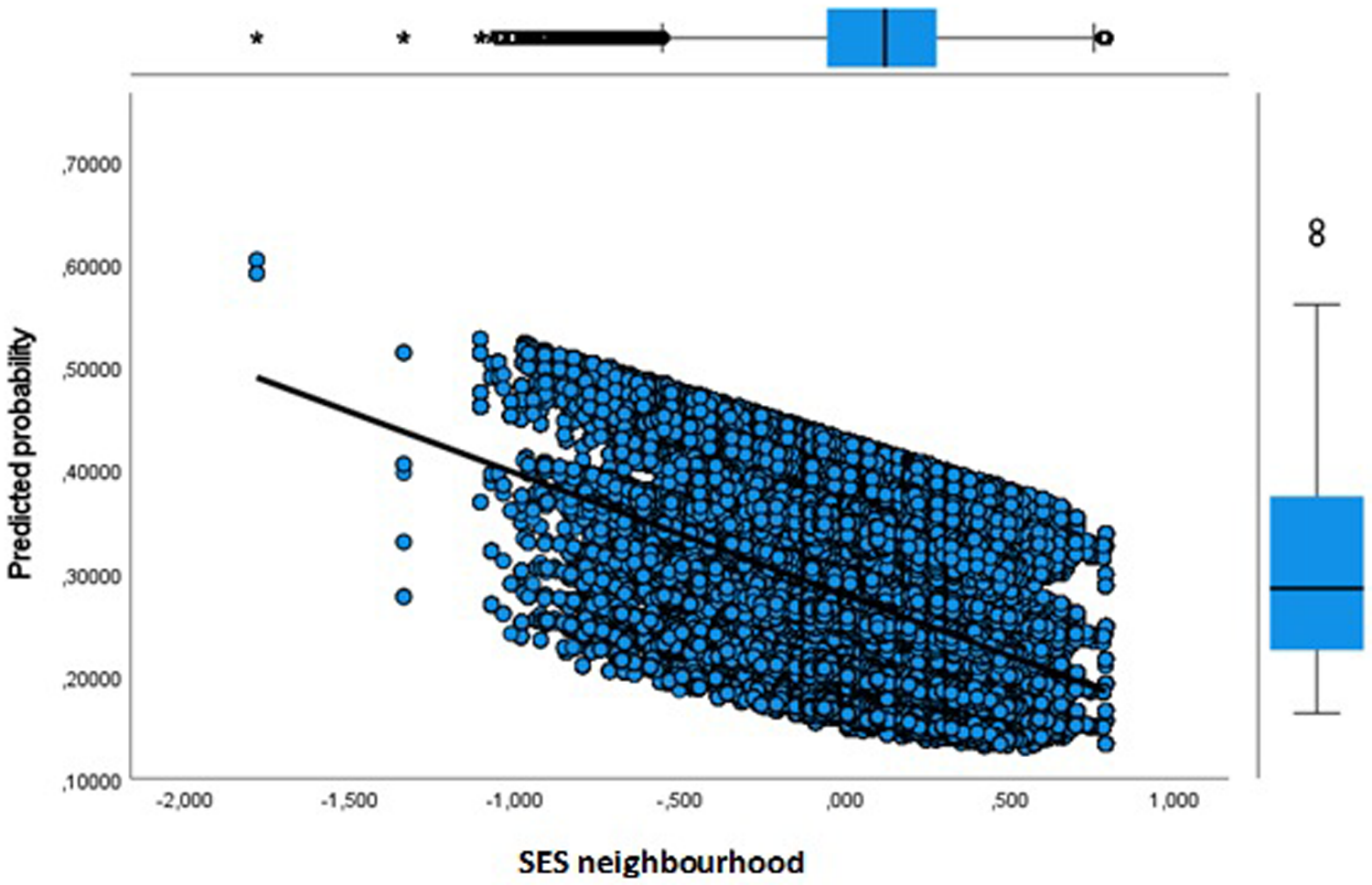
Adult dropout predicted probability by socioeconomic status of neighbourhood.

We performed several analyses. First, we conducted descriptive analyses examining differences in dropout in sport club membership between the baseline (T0 = 2019 – before COVID-19) and the COVID-19 period (T1 = 2021 - during COVID-19) for age groups and by socioeconomic status of the neighbourhood (Figures 1, 2). Second, we employed logistic regression analyses separately for youth and adults (Tables 1, 2) as we anticipate that youth have to rely to a larger extent on their nearby social and physical environment. In a first step of our logistic regression, we estimated a baseline model containing age and urbanity of a member's area of residence only. In a second step, we introduced the SES of the neighbourhood (baseline and social environment). This model allowed us to investigate whether geographical variation in dropout is explained by SES of the neighbourhood. In a third step, we included aspects of the physical environment, being our measures of availability of sport facilities and physical activity friendliness. In a fourth step, we estimated a full model with all characteristics included. The estimates of the full model are used to calculate predicted probabilities for dropout and we visualized these in Figures 3–8. For the figures we used interval scores for our explanatory variables (instead of quintiles).

**Table 1:** Descriptives of variables.

	Minimum	Maximum	Mean	Std. Deviation
All age groups (*N* = 3.614.875)				
Dropout in 2021	0.00	1.00	0.27	0.45
Age group	1.00	9.00	4.39	2.49
Urbanity	0.00	4.00	2.23	1.23
SES neighbourhood	−1.78	0.79	0.10	0.26
Sport facilities - KISF	0.00	100.00	22.36	13.83
Physical activity friendliness - KIPAF	6.00	94.00	62.57	14.47
Youth (4–18 years) (*N* = 1.103.066)				
Dropout in 2021	0.00	1.00	0.28	0.45
Age group	1.00	2.00	1.42	0.49
Urbanity	0.00	4.00	2.25	1.22
SES neighbourhood	−1.11	0.79	0.12	0.26
Sport facilities - KISF	0.00	100.00	22.04	13.58
Physical activity friendliness - KIPAF	6.00	91.00	62.79	14.33
Adults (18 years and older) (*N* = 2.511.809)				
Dropout in 2021	0.00	1.00	0.27	0.44
Age group	3.00	9.00	5.69	1.80
Urbanity	0.00	4.00	2.22	1.23
SES neighbourhood	−1.78	0.79	0.10	0.26
Sport facilities - KISF	0.00	100.00	22.50	13.93
Physical activity friendliness - KIPAF	6.00	94.00	62.47	14.53

**Table 2:** Logistic regression analysis of sports club dropout between 2019 and 2021 for youth (4–18 years; *N* = 1.103.066).

			Model 1			Model 2			Model 3			Model 4
	Exp(B)		S.E.	Exp(B)		S.E.	Exp(B)		S.E.	Exp(B)		S.E.
Age (ref. = 4–13 years)	1.244	***	0.004	1.254	***	0.004	1.247	***	0.004	1.255	***	0.004

Hardly urbanized	1.037	***	0.009	1.063	***	0.009	1.013		0.009	1.047	***	0.009
Moderately urbanized	1.103	***	0.009	1.150	***	0.009	1.036	***	0.009	1.106	***	0.009
Strongly urbanized	1.211	***	0.008	1.224	***	0.009	1.087	***	0.009	1.140	***	0.009
Extremely urbanized	1.310	***	0.009	1.246	***	0.009	1.134	***	0.010	1.138	***	0.010
SES neighbourhood. (ref.=bottom quintile)												
2nd quintile				0.805	***	0.007				0.812	***	0.007
3rd quintile				0.717	***	0.007				0.724	***	0.007
4th quintile				0.644	***	0.007				0.654	***	0.007
top quintile				0.580	***	0.007				0.589	***	0.007
Sport facilities - KISF (ref.=bottom quintile)												
2nd quintile							0.973	***	0.007	0.948	***	0.007
3rd quintile							0.941	***	0.007	0.921	***	0.007
4th quintile							0.913	***	0.007	0.890	***	0.007
top quintile							0.875	***	0.007	0.869	***	0.007
Physical activity friendliness - KIPAF (ref.=bottom quintile)												
2nd quintile							1.088	***	0.007	1.044	***	0.007
3rd quintile							1.093	***	0.007	1.028	***	0.007
4th quintile							1.125	***	0.007	1.042	***	0.007
top quintile							1.262	***	0.007	1.092	***	0.008
Constant	0.316	***	0.008	0.426	***	0.009	0.325	***	0.011	0.460	***	0.012

## 5. Results

To determine whether there is age variation and spatial distribution of dropout during COVID-19, descriptive results are presented in Figure 1. Our results show that dropout is highest among the 25–35 years old, and lowest among those between 55 and 75 years. In Figure 2 we display dropout by SES neighbourhood illustrating that the dropout for both youth and adults is highest in the lower SES quintiles.

To test our hypotheses, we consider whether the differences in dropout might be explained by (1) age and urbanity, (2) socioeconomic status of the neighbourhood and (3) physical environment of the neighbourhood (key indicators on a neighbourhood level). Table 2 presents dropout estimates of a logistic regression analyses for youth (4–18 years), and Table 3 presents estimates for adults (18 years and older). Exp(B) coefficients represent the effect size of the factors included in the model and give information about effect direction. An Exp(B) greater than 1 indicates a positive effect, while an Exp(B) less than 1 indicates a negative effect (48).

**Table 3:** Logistic regression analysis of sports club dropout between 2019 and 2021 for adults (18 years and older; *N* = 2.511.809).

			Model 1			Model 2			Model 3			Model 4
	Exp(B)		S.E.	Exp(B)		S.E.	Exp(B)		S.E.	Exp(B)		S.E.
Age groups (ref. = 18–25 years)												
25–35 years	1.074	***	0.005	1.057	***	0.005	1.071	***	0.005	1.056	***	0.005
35–45 years	0.679	***	0.005	0.679	***	0.005	0.677	***	0.005	0.679	***	0.005
45–55 years	0.483	***	0.005	0.489	***	0.005	0.483	***	0.005	0.489	***	0.005
55–65 years	0.379	***	0.005	0.382	***	0.005	0.379	***	0.005	0.382	***	0.005
65–75 years	0.381	***	0.006	0.380	***	0.006	0.381	***	0.006	0.381	***	0.006
75 year and older	0.662	***	0.007	0.655	***	0.007	0.662	***	0.007	0.657	***	0.007
Urbanity (ref. = not-urbanized)												
Hardly urbanized	1.050	***	0.006	1.062	***	0.006	1.040	***	0.006	1.057	***	0.006
Moderately urbanized	1.098	***	0.006	1.115	***	0.006	1.069	***	0.006	1.099	***	0.006
Strongly urbanized	1.229	***	0.006	1.221	***	0.006	1.170	***	0.006	1.186	***	0.006
Extremely urbanized	1.431	***	0.006	1.378	***	0.006	1.344	***	0.007	1.330	***	0.007
SES neighbourhood (ref. = bottom quintile)												
2nd quintile				0.885	***	0.005				0.885	***	0.005
3rd quintile				0.837	***	0.005				0.837	***	0.005
4th quintile				0.790	***	0.005				0.792	***	0.005
top quintile				0.757	***	0.005				0.758	***	0.005
Sport facilities - KISF (ref. = bottom quintile)												
2nd quintile							0.966	***	0.005	0.958	***	0.005
3rd quintile							0.957	***	0.005	0.951	***	0.005
4th quintile							0.929	***	0.005	0.923	***	0.005
top quintile							0.923	***	0.005	0.928	***	0.005
Physical activity friendliness - KIPAF (ref. = bottom quintile)												
2nd quintile							1.037	***	0.005	1.013	**	0.005
3rd quintile							1.055	***	0.005	1.019	***	0.005
4th quintile							1.047	***	0.005	1.006		0.005
top quintile							1.081	***	0.005	1.009		0.005
Constant	0.488	***	0.006	0.574	***	0.007	0.507	***	0.008	0.610	***	0.008

Our baseline model for both youth and adults only include age and urbanity and underscores that people living in more urbanized areas were more likely to dropout than people living in less urbanized areas. In Model 2 it is shown that a person's neighbourhood SES is important in explaining dropout for both youth and adults. For youth living in advantageous SES neighbourhoods, it is far less likely to dropout during COVID-19 compared to those living in lower SES neighbourhoods [Exp(B) = 0.580]. Moreover, adults living in a higher SES neighbourhood also have lower odds of dropping out as a sport club member compared to those living in lower SES neighbourhood [Exp(B) = 0.757].

Next, in Model 3 we separately included physical environment features of the neighbourhood to address the issue whether the sport facility provision and a physical activity friendly environment affects the likelihood of dropout. Our results show that more sport facilities (KISF) in a neighbourhood indeed lead to a lower likelihood of dropout among youth and adults. Physical activity friendliness (KIPAF) shows the opposite; the more friendly a neighbourhood is in terms of options to be active in the public space, the more likely it is that young inhabitant's dropout during COVID-19. For adults, the results for physical activity friendliness are less pronounced.

Finally, Model 4 in which we include all aspects simultaneously shows equivalent results. Only the estimates for physical activity friendliness of the neighbourhood were somewhat smaller in the full model. Again, for youth the SES of their neighbourhood plays a key role in explaining dropout, with youth living in the lowest SES neighbourhoods having the highest risks of dropout. In addition, the older youth are more likely to dropout compared to the 4–13 years olds [Exp(B) = 1.255]. We also observed smaller differences for urbanity when adding information on the SES of the neighbourhood and the physical environment. But still, the odds for dropout are highest in the most strongly urbanized areas [Exp(B) = 1.140], and extremely urbanized areas [Exp(B) = 1.138]. Youth living in areas with abundant sport facilities seem to have a lower likelihood of dropout [Exp(B) = 0.869].

For adults results are similar, although the effects of neighbourhood level seem smaller. Probably, this indicates that adults are to a lesser extent influenced by their immediate social and physical environment maybe because of more mobility options compared to that of youth. In the full model the variation by age is most pronounced. It further shows that adults living in extremely urbanized areas have a relatively high likelihood of dropout [Exp(B) = 1.330]. Also, the likelihood of dropout decreases with an increase in the SES of a person's neighbourhood. The same holds true for having more sport facilities in a neighbourhood, although the differences are relatively small.

In Figures 3–8 we visualized predicted probabilities for dropout from the full model, both for youth and adults, for our neighbourhood measures of the SES and the physical environment.

## 6. Discussion

Prior research often focused on differences in sport participation and these studies repeatedly found that sport participation is socially stratified (8, 49). People from the higher socioeconomic strata are more likely to practice sport than those from the lower socioeconomic strata. Few studies however have focused on inequality in dropout of sport participation (see for an exception 50). When focusing on tackling inequality in sport participation it however seems especially important to direct attention to retaining vulnerable groups among sport participants. For this reason, in this article we address dropout in sport club membership during COVID-19 in the Netherlands. We consider this to be crucial in relation to the often-mentioned beneficial aspects of sport participation particularly under the conditions of this pandemic. Recent studies already provided evidence that vulnerable groups are impacted the most by crises (34), and demonstrated increased inequality in sport participation during the COVID-19 pandemic (23, 24).

To deal with dropout in sport club membership under COVID-19 we especially focused on the neighbourhood level. In doing so we provide insights in the spatial distribution of dropout from voluntary sport clubs in the Netherlands between 2019 and 2021. Our results reveal that the socioeconomic status of the neighbourhood is important in the explanation of an individual's dropout from a sport club; both youths and adults living in low status neighbourhoods are most likely to dropout during COVID-19. Regarding the physical environment, it is established that a more divers opportunity structure for sport club membership increases the likelihood that individuals in these environments uphold their membership. Contrarily, a more physical activity friendly environment increases the likelihood of dropping out. In a comparison it stands out that indicators of the physical environment show substantially smaller odds ratios compared to the indicator of the SES, indicating that a neighbourhood's social economic composition is most important. This is in line with earlier findings on the rural-urban divide in sport participation in the Netherlands (7).

Several studies also indicate that there are difficulties of returning to sport after the COVID-19 lockdown. This seems to hold true for sport participation in general, but also for organized sports (51, 52). Individual's opportunities to return to sport practices likely differ based on difference in forms of capital they have at their disposal. Consequently, scholars emphasized that the sport sector should prioritize on the inclusion within community sport (53), and could benefit from an even larger focus on low socioeconomic groups (24). Our outcomes support this call to action. Based on registration data we observed a clear peak in dropout in the lowest socioeconomic neighbourhoods.

Apart from attention for low SES groups in low SES neighbourhoods, we recommend policy makers to invest in a support system for the community sport clubs within neighbourhoods. It is known from previous research that vulnerable sport clubs are more likely to be situated in lower socioeconomic status neighbourhoods, while stable and financially sound sport clubs are found more often in high status neighbourhoods. Consequently, it may be assumed that differences in dropout between neighbourhoods is partly related to the organizational effectiveness of the voluntary sport clubs themselves. This links closely with conclusions of Staley et al. (51: 18) who noted that the “community sport club environment is complex, and the perceived challenges in returning to sport after the COVID-19 shutdown were multifaceted and context-specific”. And, to previous studies that emphasized that a stable commitment of members to sport clubs is not only the outcome of individual characteristics. It is also affected by club-specific structural conditions (54). More research into the trajectories of voluntary sport clubs and the related development of membership statistics is recommended.

A broad study on organizational legitimacy of voluntary leisure organisations already showed that the geographical distribution of resources is uneven and that in low status neighbourhoods survival rates are lower (55). During the COVID-19 pandemic an ethnographic study in the Netherlands illustrated different trajectories of two community sport clubs, one in a high status neighbourhood and one in a low status neighbourhood (36). This study indicated clear differences both in the offerings of these two community sport clubs, as well as in the dropout of members. Where in the low status neighbourhood the sport club had troubles organizing activities and saw a relatively large share of their members dropping out during COVID-19, the opposite seemed true for the high SES sport club (36). These prior studies support our recommendation to offer more support to voluntary sport clubs in lower SES neighbourhoods as capabilities or structural conditions of sport clubs in high and low SES neighbourhoods might had an influence on the higher dropout rates found in the lower SES neighbourhoods.

Some limitations of our study must be mentioned. First, we have little information on individual characteristics of the sport club members. This sure is a disadvantage because compositional differences might explain part of the found neighbourhood effects. For privacy reasons NOC*NSF provided limited access to socio-demographics in combination with the specific information on a member's neighbourhood. From previous studies we know that gender differences in sport participation are prominent (56), and this raises the question how the sport participation of men and women developed during COVID-19. Future studies might want to dig deeper in this issue. Second, the KISS data provided no information on the frequency of sport participation; only number of sport club memberships was available. Since theoretically membership does not equal participation, it might be that our studies overrate sport club participation. Because a person could be a member during COVID-19 without actively engaging in sports.

Moreover, it is appropriate to note that our paper addresses dropout from sport club membership. It is not certain that ending a (paid) membership corresponds with quitting sport participation entirely. A voluntary sport club membership may be substituted with a commercial fitness centre and also sporting outside a sport club is possible, for instance in public spaces. The found positive impact of a physical activity friendly environment on dropout provides support for this presumption; opportunities for sports in the public space likely have provided sport participants with alternatives for their sport club membership. This is in line with the general tendency that most European countries report a trend towards more sport practice in public space at the cost of club membership (21).

This tendency to more individual sport participation and less club-based sport participation is visible in the Netherlands as well. Although, within sport policy the voluntary sport clubs hold a central position. In comparison with other European countries there is relatively much policy attention for sport clubs (3). With the more diffuse market of sport providers local policy makers also turn to other sport providers outside of the realm of voluntary sport clubs. Within the current sport policy document (57) also commercial sport providers are positioned as a relevant policy partner alongside the National Sport Federation (NOC*NSF). However, membership of voluntary sport clubs remains an important sport policy indicator in the Netherlands and participating and volunteering within voluntary sport clubs is highly valued. With this the increased inequality in sport club participation is contrary to general policy intentions and does require a reorientation on the possible instruments to utilize.

Our study also has implications for developments in the inequality of sport participation. Studies on inequality in sport participation in the Netherlands illustrate that lower educated and people with financial difficulties were more likely to quit practicing sport during COVID-19 (23). Furthermore, these groups were also less likely to bounce back to sport participation after the COVID-19 pandemic (58). Our findings on more dropout in low SES neighbourhoods point in a similar direction and urges for policy measures to backfire the growing divide in sports. Based on surveys on overall sport participation in 2022 we know that vulnerable groups are less likely to bounce back (58). We anticipate that this could also be the case for sport club membership in 2022 or 2023. Consequently, levels of inequality in sport club participation are likely to rise further.

Policy makers should be aware of the disadvantaged position of particularly youth in lower SES neighbourhoods. More attention for sport promotion in these areas is much needed with an eye on possible barriers to sport club participation. Subsidies to cover costs of sport club membership for the financially deprived are in place in most municipalities, but somehow this does not seem to be sufficient. On a national level a subsidy program is implemented for municipalities to appoint neighbourhood sport coaches dedicated to lower socioeconomic status neighbourhood. All these measures until now do not seem to have the intended effect. Within the Netherlands the relatively low sport participation in lower SES neighbourhoods is identified as one of the six wicked problems in sport policy (59). Additional research is needed on the perspectives of the vulnerable groups and the role sport participation plays in their lives. In addition, support systems for voluntary sport clubs should target these neighbourhoods and contribute to vital and strong sport club providers in low SES neighbourhoods. The results show that a more divers sport supply and as such better opportunity structure for sport club participation decreases the likelihood of dropout. An interesting avenue for further study would be the presence of sport facilities and voluntary sport clubs in low status neighbourhoods. A previous study on the distribution of sport facilities in the Netherlands with data from 2014 did not support the idea of deprivation amplification in which areas with poorer people would have inferior public and private sport facilities (30). Although a limited variety of sport facilities was visible in lower SES neighbourhoods. However, the question is to what extend these sport facilities and the voluntary sport clubs that uses these facilities have survived the past years with financial difficulties, a COVID-pandemic, and the energy crisis.

## 7. Conclusions

The COVID-19 measures have made a clear impact on the sport sector and on sport participation of individuals. Our study shows that in the Netherlands a substantial amount of sport club members dropped out between 2019 and 2021. We showed with our research using a socio-ecological perspective that this dropout is affected by contextual aspects at the neighbourhood level. Especially, living in a low SES surrounding seems to negatively influence membership of a sports club during COVID-19. We also found that a good opportunity structure of abundant sport facilities in a neighbourhood helps to remain a member.

Our study illustrates the importance of contextual influences at the neighbourhood level utilizing population registration data of intra-personal development of sport club membership. With this innovative approach we add to the rather inconclusive literature so far on how sport club membership developed over the course of the COVID-19 pandemic. However, this population registration data comes at the price of limited detail. It would be interesting to further investigate individual characteristics and data of voluntary sport clubs where dropout occurs to better understand individual processes and dynamics underlying the dropout.

Nevertheless, based on our outcomes we support a more geographical focus in sport promotion policies, directed both at individuals and at voluntary sport clubs in low SES neighbourhoods. To end, given the relatively high degree of dropout, special attention should be paid to the retention of sport club members within voluntary sport clubs.

## Data Availability

The data analyzed in this study is subject to the following licenses/restrictions: The KISS-data was provided for the purpose of this ZonMw project only. Requests to access these datasets should be directed to remco.hoekman@ru.nl.

## Footnotes

^1^www.sportenbewegenincijfers.nl/kernindicatoren for an overview of key indicators of sport policy in the Netherlands.

^2^In a robustness analysis we compared dropout in the original full dataset with the final dataset (without missings). In both datasets we found equal dropout percentages (27%).
